# Extracorporeal Shock Wave Rebuilt Subchondral Bone In Vivo and Activated Wnt5a/Ca^2+^ Signaling In Vitro

**DOI:** 10.1155/2017/1404650

**Published:** 2017-10-15

**Authors:** Lai Yu, Shuitao Liu, Zhe Zhao, Lin Xia, Haochong Zhang, Jing Lou, Jun Yang, Gengmei Xing, Gengyan Xing

**Affiliations:** ^1^Xiamen Chang Gung Hospital, Xiamen, Fujian, China; ^2^Key Lab for Biomedical Effects of Nanomaterials and Nanosafety, Institute of High Energy Physics, Chinese Academy of Sciences, Beijing, China; ^3^The Fourth Military Medical University, Xi'an, Shaanxi, China; ^4^General Hospital of Chinese People's Army Police Force, Beijing, China

## Abstract

**Background:**

This study aimed to identify the optimal extracorporeal shock wave (ESW) intensity and to investigate its effect on subchondral bone rebuilt* in vivo* and Wnt5a/Ca^2+^ signaling* in vitro* using an osteoarthritis (OA) rat model and bone marrow mesenchymal stem cells (BMMSCs), respectively.

**Methods:**

OA rats treated with (OA + ESW group) or without (OA group) ESW (*n* = 12/group) were compared with healthy controls (control group, *n* = 12). Gait patterns and subchondral trabecular bone changes were measured. Western blot and quantitative real-time polymerase chain reaction detected protein expression and gene transcription, respectively.

**Results:**

The gait disturbances of OA + ESW group were significantly improved compared with the OA group at 6th and 8th weeks. The micro-CT analysis indicated that the BMD, BSV/BV, BV/TV, Tr.S, and Tr.Th are significantly different between OA group and OA + ESW group. Expression of Wnt5a was increased rapidly after ESW treatment at 0.6 bar and peaked after 30 min.

**Conclusions:**

ESW were positive for bone remodeling in joint tibial condyle subchondral bone of OA rat. ESW prevented histological changes in OA and prevented gait disturbance associated with OA progression. Optimal intensity of ESW induced changes in BMMSCs via activation of the Wnt5a/Ca^2+^ signaling pathway.

## 1. Introduction

Osteoarthritis (OA) is the most common arthritis and is characterized by chronic joint pain and stiffness. The underlying cause of OA is thought to be imbalanced metabolism of cartilage and subchondral bone [1, 2]. OA can severely impact patients' ability to work and carry out normal daily activities. To improve OA clinical symptoms, repair and reconstruction of the damaged cartilage and subchondral bone is essential [3]. In the restructuring process, osteoblast precursors (bone marrow mesenchymal stem cells, BMMSCs) first migrate from the bone marrow compartment to the bone surface and adhere, where they undergo differentiation and deposit bone matrix [4, 5]. Some researchers have reported that Wnt/beta-catenin and Wnt/calcium (Ca^2+^) signaling pathways play important roles in regulating BMMSC differentiation [6–8]. Wnt5a/Ca^2+^ signaling was previously shown to inhibit the self-rebuild of BMMSCs and stimulating osteogenic differentiation [7].

The application of an extracorporeal shock wave (ESW) is an emerging noninvasive treatment for osteopathy [9–11]. This novel form of mechanical stimulation promotes osteogenesis [12], thus inhibiting osteoclast activity and cartilage degeneration during bone remodeling [13, 14]. After short- or long-term treatment, positive effects of ESW on osteogenic activation through osteoblast differentiation and proliferation have been reported* in vitro* and* in vivo*, but the underlying mechanism remains unclear [15]. Clinical symptoms of OA rats were also reported to improve with ESW treatment (ESWT) [8]. We therefore hypothesized that ESW treatment (ESWT) can regulate the Wnt/Ca^2+^ signaling pathway to promote BMMSCs osteogenic differentiation, thus improving clinical symptoms of OA rats. However, the effect of shock waves on Wnt5a/Ca^2+^ signaling pathway for osteoblasts has not been reported [16].

The present study examined the therapeutic effect of ESW in OA rats using microcomputed tomography (*µ*CT) measurements, histological assessments of tissue damage, and gait analysis. Further, semiquantitative real-time polymerase chain reaction (Q-RT-PCR) and western blotting were also used to assess the expression and distribution of Wnt5a and other proteins in treated BMMSCs.

## 2. Method

### 2.1. Animals

Thirty-six 8-week-old Sprague Dawley male rats (170–190 g) were used. All animal experiments were approved by the ethical committee of the university laboratory and performed in strict accordance with the institutional guidelines. The animals were housed at 23 ± 1°C under a 12-hour light-dark cycle with free access to food and water. A week of acclimation was allowed prior to the start of the experiment. Twelve rats were randomly selected for control group (Ctrl), and the other 24 were used to shape OA model. 50 *μ*l sodium monosodium iodoacetate (MIA) saline solution (20 *μ*g/*μ*l) was injected into the articular cavity through the patellar ligament [17, 18] of the rats, and the Ctrl was injected with the same volume of 0.9% saline under identical conditions. Two weeks later, the model animals were randomly divided into two groups. Anesthetized OA rats by hypothermic 1.5% chloral hydrate in one group were treated by ESW (OA + ESW) with suitable energy (1.0 bar, 6 Hz, and 800 s), and the sham animals (OA) received pseudo shock wave (sound without energy). All treatments were given once a week. After awakening, the rats were returned to their home cage.

### 2.2. Gait Analysis

Gait analysis used the CatWalk XT (the CatWalk XT was provided by Medical School of Chinese PLA) (Noldus, Netherlands), which consists of a glass plate runway equipped with a fluorescent tube and a camera. Gait analyses were carried out at baseline and at weeks 2, 4, 6, and 8 as described previously [19].

### 2.3. Subchondral Bone Measurements on Micro-CT Scans

After anesthetization in week 8, all knees of treated rats were scanned using micro-CT (the micro-CT was provided by Institute of Laboratory Animal Sciences, CAMS & PUMC) (Inveon CT, SIEMENS, Munich, Germany) with voltage 70 kV, current 142 A, exposure time 1475 ms, 1-mm aluminum filter, 0.5° rotation steps, and 18 *μ*m resolution. All data sets were segmented with a local threshold algorithm. The obtained projection of the image is using the modified Feldkamp back projection for reconstruction. The separation of cortex and bone was performed with the built-in software. Bone trabeculae thickness and volume were measured at the epiphysis of the tibia. BV/TV represents the ratio of trabecular bone volume (BV, in mm^3^) and tissue volume (TV, in mm^3^).

### 2.4. Immunohistochemistry and Histomorphometry Analysis

After treated rats were sacrificed, the intact knees were rapidly removed and fixed in 4% formaldehyde solution for 48 h, followed by decalcifying in 10% EDTA (pH 7.4) solution for 3 weeks. The tissues were then dehydrated with the ASP200S hydroextractor (all the other instruments were supported by Key Lab for Biomedical Effects of Nanomaterials and Nanosafety, Institute of High Energy Physics) (LEICA, Wetzlar, Germany) and embedded with paraffin. Joint compartments of the lateral were vertically cut into 4 *μ*m thick sections on a microtome (Leica RM2235) and stained with hematoxylin and eosin (HE). The Mankin score of OA pathological grading was executed according to the criteria in Table S1. After xylene dewaxing, gradient ethanol hydration, hydrogen peroxide blocking, and antigen retrieval, the sections were incubated with a primary antibody to Wnt5a (Abcam, Cambridge, UK) for 12 hours, followed by a horseradish peroxidase- (HRP-) labeled secondary antibody. Positive staining was observed and recorded by microscope, and the areas were picked out by randomly selecting six areas in three sections of the same specimen. All images were captured with a Cool CCD camera (SNAP-Pro c.f. Digital kit; Media Cybernetics, Silver Spring, MD, USA) and analyzed using Image-Pro Plus 6.0 image-analysis software (Media Cybernetics). The percentages of the positive cells were counted in each region of the subchondral bone.

### 2.5. BMMSCs

Isolating and culturing of BMMSCs are described in the supplemental information, in Supplementary Material available online at https://doi.org/10.1155/2017/1404650. Briefly, second-generation BMMSCs were inoculated in 6-well plates (2 × 10^4^ cells/cm^2^) and treated with different energy levels of ESW after cell confluence reached about 90%. The parameters for ESWT were 1000 s, 6 Hz, and then 800 s. We set up three energy levels (0.2, 0.4, and 0.6 bar) and five time points (5 min, 15 min, 30 min, 1 h, and 3 h). In the control group, the treatment was the same except for ESWT.

### 2.6. Western Blot

After ESWT, cells were harvested by centrifugation and washed 3 times with phosphate-buffered saline (PBS); then total protein in the cells was extracted and quantified as previously described [7]. Using standard procedures, total proteins were separated by 10% sodium dodecyl sulfate- (SDS-) polyacrylamide gel electrophoresis and transferred onto nitrocellulose membranes. Nonspecific sites were blocked with 5% fetal bovine serum (FBS) in Tris-buffered saline (TBS) with 0.1% Tween-20. The primary antibodies including rabbit polyclonal anti-Wnt5a (ab174100), anti-PKC (ab19031), anti-PLC (ab185724), anti-CaMKII (ab34703) (Abcam), and anti-GAPDH (sc-25778, 1 : 200, Santa Cruz Biotechnology, Santa Cruz, CA, USA) were diluted and coincubated with the nitrocellulose membranes according to the manufacturers' instructions. The secondary antibody was IRDye 680 Goat Anti-Rabbit (Abcam). Detection and quantification were performed with photographic film (Kodak, Rochester, NY, USA) and Tanon Gis (Shanghai, China).

### 2.7. Semiquantitative Real-Time Polymerase Chain Reaction (Q-RT-PCR)

Total cellular RNA was isolated with TRIzol reagent (Invitrogen, Carlsbad, CA, USA) according to the manufacturer's instructions. First-strand cDNA was synthesized from 1 mg total RNA using an ExScript RT reagent kit (Takara Bio Inc., Shiga, Japan) following the operation manual. PCR was performed using Platinum Taq DNA polymerase (Invitrogen) in a PCR Thermal Cycler Dice (Takara Bio) under the following conditions: 95°C for 10 s (initial denaturation), then 40 cycles at 95°C for 5 s, and 60°C for 30 s, followed by a dissociation program at 95°C for 15 s, 60°C for 30 s, and 95°C for 15 s. Primer sequences, annealing temperatures, cycle number, and product sizes for Wnt5a, phospholipase C (PLC), protein kinase C (PKC), and calcium/calmodulin-dependent protein kinase II (CaMKII) are shown below. Glyceraldehyde 3-phosphate dehydrogenase (GAPDH) was used as an internal standard. Expression levels of target genes were calculated using the 2^−ΔΔct^ values.

Primer sequences were as follows (Invitrogen): Wnt5a: forward AAGCAGGTCGCAGGACAGTA and reverse CGCTGCGCTGTCATACTTCT; PLC: forward GAGGAAGTGGCCAAGGAATG and reverse AGAGGAATGCGCCCTTCTG; PKC: forward GGCCTGCAATGTCAAGTCTG and reverse GCTGTGCAGACGGAACTTGT; CaMKII: forward TATCGTCCGACTCCATGACAG and reverse GTAGCACAGCCTCCAGGATCT; GAPDH: forward GCATCCTGCACCACCAACT and reverse GCAGTGATGGCATGGACTGT.

### 2.8. Immunofluorescent Assay

Thirty minutes after ESWT, cells cultured in glass-bottom dishes were washed three times with PBS and fixed with 4% paraformaldehyde for 10 minutes. Then cells were permeabilized in 10% BSA and 3% Triton X100 and incubated overnight at 4°C with anti-Wnt5a (ab174100, Abcam) dissolved in PBS with 3% BSA. After washing in PBS, cells were incubated with IgG H&L (Texas Red®) Goat Anti-Rabbit (ab6719, Invitrogen/Life Technologies) for 2 h at room temperature in dark and stained for 15 minutes with Hoechst 33342 (H1399, Invitrogen/Life Technologies). Negative controls were prepared by omitting the primary antibody. Slides were analyzed with a Nikon confocal fluorescence microscope (Nikon, Tokyo, Japan) equipped with multispectral imaging system (PerkinElmer, Waltham, MA, USA).

### 2.9. ESW Treatment

For in vitro shock wave treatment, an unfocused pneumatic ballistic device was used (EMS Swiss DolorClast, Germany). BMMSCs of the third generation were inoculated in 6-well plates and were treated with different energy levels of ESW after cell confluence reached about 90%. The intervention parameter of ESW was 1000 s, 6 Hz for BMMSCs, and then 800 s. We set up 3 energy levels (0.2, 0.4, and 0.6 bar) and 5 time points (5 minutes, 15 minutes, 30 minutes, 1 hour, and 3 hours), respectively. In the control group, the treatment was the same except for ESW intervention.

### 2.10. Statistical Analysis

SPSS 19.0 software (SPSS Inc., Chicago, IL, USA) was used for statistical analysis. Data are expressed as mean ± standard deviation (SD). One-way analysis of variance (ANOVA) was carried out to compare the differences of means when three or more groups were assessed. For gait analysis and micro-CT images, the quantification of parameters was performed three times in a blinded fashion. Agreement for all the parameters was achieved among the three observers. We used the means of the three measurements for statistical analyses. *P* < 0.05 was considered statistically significant.

## 3. Results

### 3.1. Effect of ESWT on Gait Parameters

**Figure 1:**
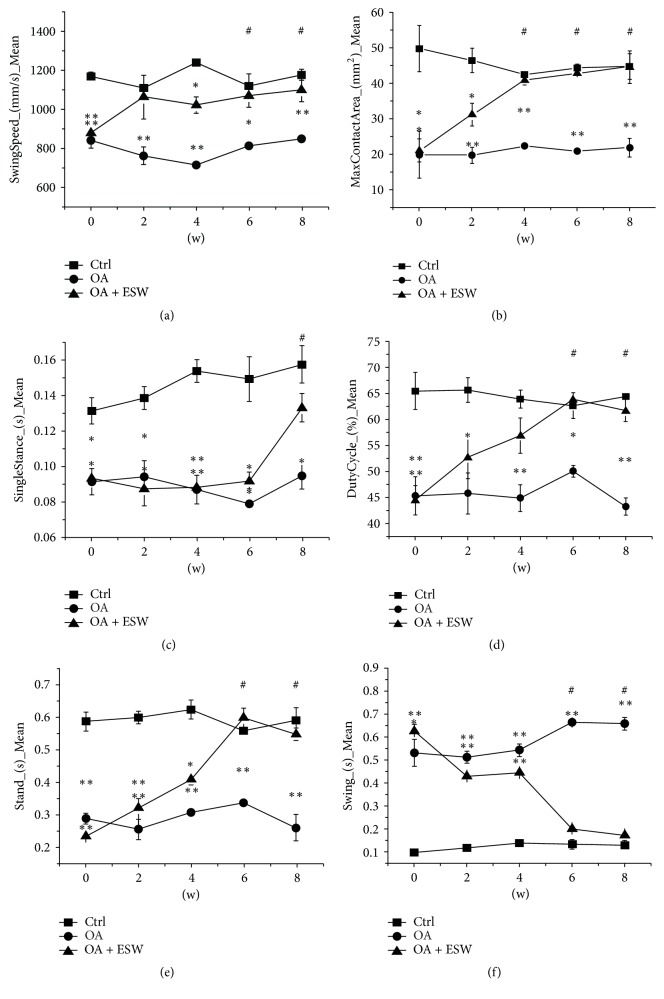
*Longitudinal changes of gait parameters in rats*. Imbalance in various indicators according to depth at 0th week (0 w), 2nd week (2 w), 4th week (4 w), 6th week (6 w), and 8th week (8 w) was measured by the gait analysis paradigm in rats after OA models were established successfully. Values presented are mean ± 95% confidence interval of the corresponding numerical. In the OA and OA + ESW group, significant differences were seen compared with the control. (a) The swing speed (mm/s) at 2 w, 6 w, and 8 w (*n* = 12; ^*∗*^*P* < 0.05 and ^*∗∗*^*P* < 0.01 compared with the control group). (b) The max contact area (mm^2^) at 4 w, 6 w, and 8 w (*n* = 12, ^*∗*^*P* < 0.05 and ^*∗∗*^*P* < 0.01 compared with the control group). (c) The single stance (s) at 8 w (*n* = 12, ^*∗*^*P* < 0.05 and ^*∗∗*^*P* < 0.01 compared with the control group). (d) The duty cycle (%) at 6 w and 8 w (*n* = 12, ^*∗*^*P* < 0.05 and ^*∗∗*^*P* < 0.01 compared with the control group). (e) The stand (s) at 6 w and 8 w (*n* = 12; ^*∗*^*P* < 0.05 and ^*∗∗*^*P* < 0.01 compared with the control group). (f) The swing (s) at 6 w and 8 w (*n* = 12, ^*∗*^*P* < 0.05 and ^*∗∗*^*P* < 0.01 compared with the control group). No difference was observed within groups but for OA + ESW groups # represents the time point at which significant differences were seen in OA + ESW group compared with the baseline (0 w). The other essential results were attached in supplemental information (Figure S3). Error bar is standard error of mean [SEM].

The gaits of rats in each group were analyzed from 0 to 8 weeks after treatment. The results showed that clinical symptoms of OA rats were significantly improved by ESWT. Compared with untreated OA rats, the positive treatment effect of ESWT appeared in the 2nd week, with the most beneficial effects from the 4th to 8th weeks (Figure 1). Compared with control, there were significant differences in swing speed (Figure 1(a)) at weeks 6 and 8; max contact area (Figure 1(b)) in weeks 4, 6, and 8; single distance (Figure 1(c)) in week 8; and duty cycle (Figure 1(d)), stand (Figure 1(e)), and swing (Figure 1(f)) in weeks 6 and 8.

### 3.2. Subchondral Plate Thickness and Bone Porosity

**Figure 2:**
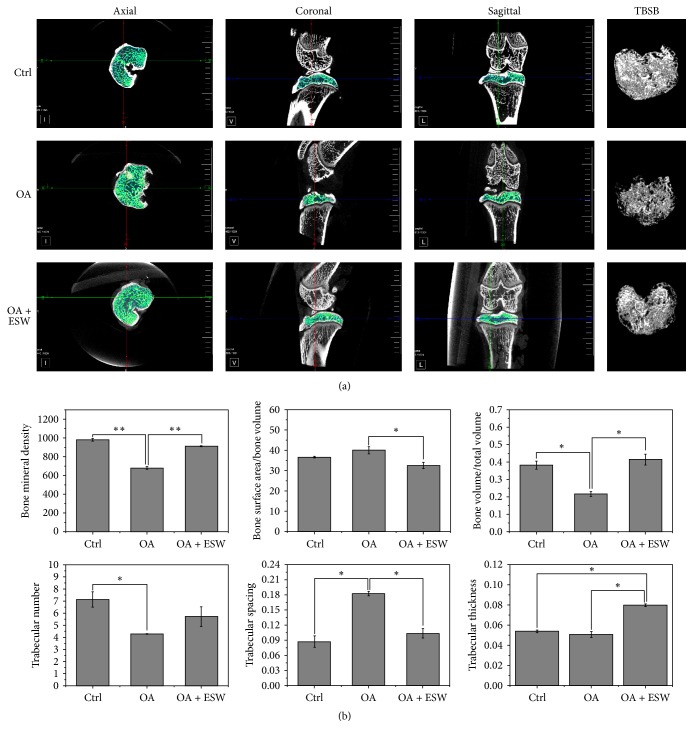
*Characterization of OA-induced subchondral bone changes in rats*. (a) TBSB = three-dimensional reconstruction of trabecular bone of the subchondral bone. Micro-CT images of the joint tibial condyle subchondral bone at 8 weeks were different in the control versus ESW stimulation groups. Structural integrity of the subchondral bone was enhanced in the OA + ESW group compared with the OA group. (b) Micro-CT analysis of subchondral bone to evaluate knee joint function after ESW treatment in the MIA-induced OA rat model. BMD, BSA/BV, BV/TV, Tr.N, Tr.S, and Tr.Th data for all groups. ^*∗*^*P* < 0.05 and ^*∗∗*^*P* < 0.01 compared with control (error bar is SEM).

Micro-CT scans revealed that bone plate thickness of the joint tibial condyle subchondral bone in OA rats was significantly increased by ESWT. This finding reflects increases in bone mineral density (BMD), bone surface area/bone volume (BSA/BV), bone volume/total volume (BV/TV), and trabecular spacing (Tr.s) in OA rats treated for 8 weeks. There was no difference in trabecular number (Tr.N) between OA and OA + ESW rats. Trabecular thickness (Tr.Th) was higher in the OA + ESW group compared to the OA group, but this was not significant (Figure 2). Bone porosity was significantly decreased in the OA + ESW group compared with the OA group. Therefore, the effect of ESWT was significant improvement in subchondral bone plate structure.

### 3.3. Histological Analysis of Subchondral Bone Plate

**Figure 3:**
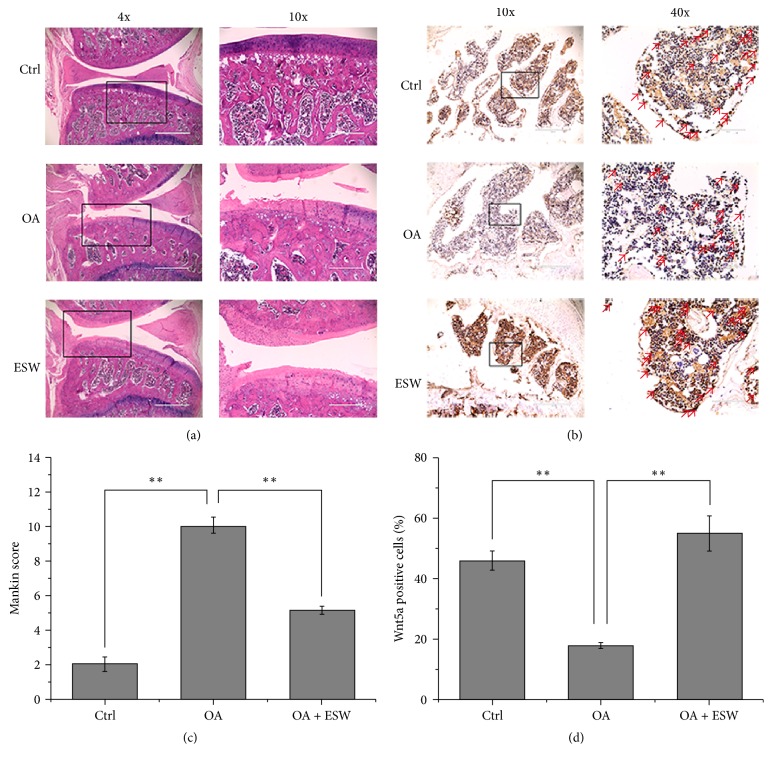
*Histopathologic assessment of cartilage and subchondral bone*. (a) Hematoxylin-eosin (HE) staining of cartilage and subchondral bone at the 8th week after OA models were established successfully. (b) Wnt5a immunohistochemical (IHC) staining of subchondral bone of knee joint tibial condyle from 8-week control or experimental groups. The red arrows represent staining positive cells. (c) The OA group showed significant increases in Mankin score that was comparable to that of control. The ESW appeared effective in OA + ESW group compared with OA group (^*∗∗*^*P* < 0.05; error bar is SEM). (d) The number of Wnt5a-positive cells within the selected area was counted. Significant decreases in Wnt5a in the OA group compared with the control. The OA + ESW group showed significant increases in Wnt5a that were comparable to the OA (^*∗∗*^*P* < 0.05, error bar is SEM).

Results from the histopathological analysis of subchondral bone plate are shown in Figure 3(a). Mankin scoring of pathological structures in the OA group were significantly higher than in OA + ESW and control rats at 8 weeks. Notably, the damage degree of articular surface of the OA + ESW group was significantly reduced compared with the OA group (*P* < 0.05, Figure 3(c)). Osteocyte lacunae were observed in the superficial zone of subchondral bone plate in OA rats, but this pathological phenomenon was attenuated by ESWT (Figure 3(a)). Wnt5a protein expression in the subchondral bone plate was immunohistochemically assessed (Figure 3(b)). Significantly increased Wnt5a labeling was induced by ESWT in OA rats (*P* < 0.05, Figure 3(d)).

### 3.4. Optimized Condition of ESWT to BMMSCs

**Figure 4:**
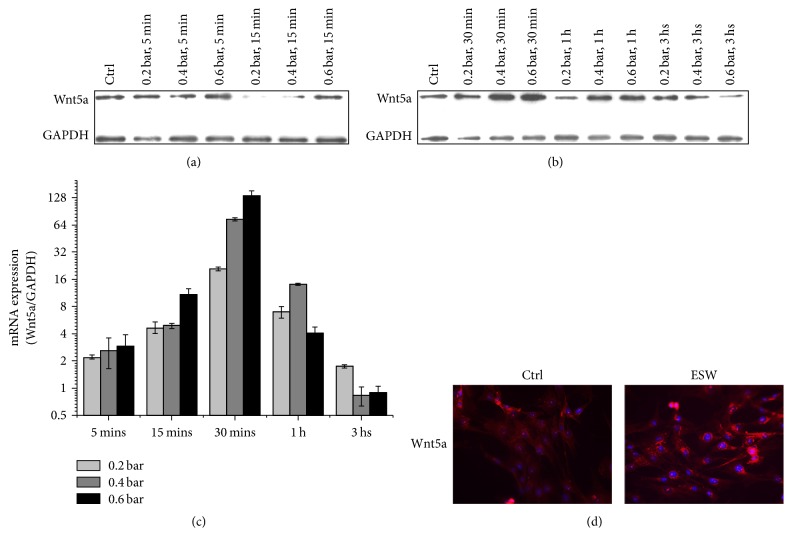
*Wnt5a expression following the intervention with various ESW energy levels*. (a, b) BMMSCs were exposed to ESW (0.2 bar, 0.4 bar, and 0.6 bar, 6 Hz, and 1000 shots) and detected at various time points as indicated. The expression of Wnt5a was analyzed by western blots. (c) BMMSCs were exposed to ESW (0.2 bar, 0.4 bar, and 0.6 bar, 6 Hz, and 1000 shots) and detected at various time points as indicated. The mRNA was extracted from the cell lysates, and Q-RT-PCR with the primers of Wnt5a and GAPDH (internal control) was performed as described in Methods. The quantitative data were shown as 2^−ΔΔct^ (*n* = 4). (d) Immunofluorescence of Wnt5a in BMMSCs. BMMSCs were treated with or without ESW (0.6 bar, 6 Hz, and 1000 s). Scale bar = 100 *µ*m. Thirty minutes after ESW intervention, cells were prepared for immunofluorescence and pictures were taken under a fluorescence microscope using a ×40 objective.

We hypothesized that ESWT induces Wnt5a protein expression in BMMSCs, which promotes their osteogenic differentiation, leading to significant improvements in the clinical symptoms of OA. We assayed Wnt5a protein expression in BMMSCs treated with different energy levels (0.2, 0.4, and 0.6 bar) for various times. An obvious effect was that ESWT induced BMMSCs to express high levels of Wnt5a protein as confirmed by western blotting. We found that 0.6 bar ESWT for 30 minutes induced the highest expression of Wnt5a in BMMSCs (Figures 4(a) and 4(b)). A variable expression trend was shown by Q-RT-PCR; Wnt5a increased and peaked (from 5 to 30 min) and then decreased and approached baseline (from 30 min to 3 h) (Figure 4(c)). In the condition, abundant fluorescent labeling of Wnt5a (red) was observed in the perinuclear space of BMMSCs (Figure 4(d)). There was a significant difference in Wnt5a fluorescent labeling between control and treated BMMSCs with 0.6 bar for 30 min.

### 3.5. Expression of Wnt5a/Ca^2+^ Signaling-Related Proteins

**Figure 5:**
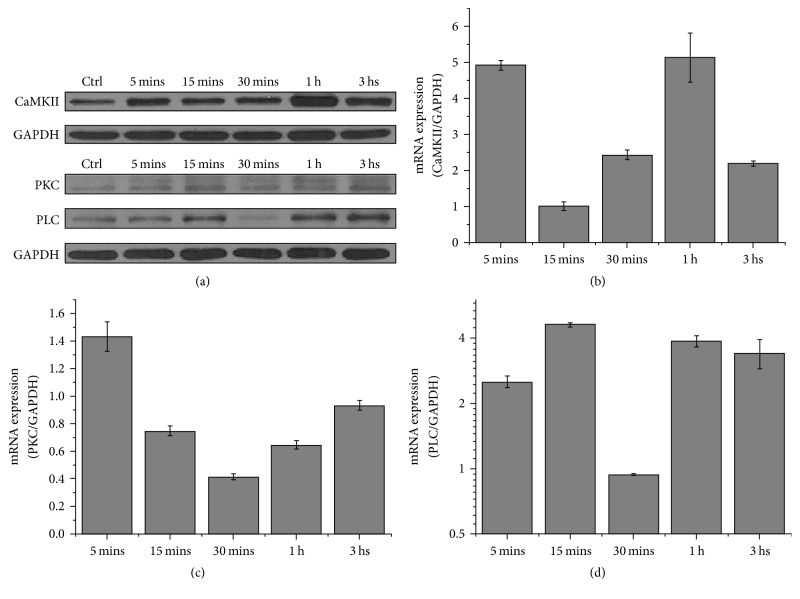
*CaMKII, PKC, and PLC expression following the intervention with various ESW energy levels*. ESW increases the mRNA levels of CaMKII and PLC and decreases the mRNA levels of PKC at different time point in BMMSCs. (a) Western blot assay of CaMKII, PKC, and PLC receptor protein expression. GAPDH served as loading control. BMMSCs were exposed to ESW (0.2 bar, 0.4 bar, and 0.6 bar, 6 Hz, and 1000 shots) and detected at various time points as indicated. (b, c, d) BMMSCs were exposed to ESW (0.2 bar, 0.4 bar, and 0.6 bar, 6 Hz, and 1000 shots) and detected at various time points as indicated. The mRNA was extracted from the cell lysates, and Q-RT-PCR with the primers of CaMKII, PKC, PLC, and GAPDH (internal control) was performed as described in Methods. The quantitative data were shown as 2^−ΔΔct^ (*n* = 4).

The expressions of Wnt5a/Ca^2+^ signaling pathway-related proteins PLC, PKC, and CaMKII in treated BMMSCs were detected by western blotting and Q-RT-PCR. CaMKII peaked at 5 min and 60 min and then returned to normal levels at 180 min (Figures 5(a) and 5(b)). Conversely, PKC expression was initially induced by ESWT and then decreased at 5 min, reached a nadir at 30 min, and returned to baseline at 180 min (Figures 5(a) and 5(c)). PLC expression was lowest with ESWT (Figures 5(a) and 5(d)). The expression levels of these genes varied but correlated with Wnt5a/Ca^2+^ signaling pathway activation following ESWT.

## 4. Discussion

OA research [20] suggests that cancellous bone collapse mass density shrinkage and subchondral bone reduction might be initiating factors in disease pathogenesis [21, 22]. The two proposed goals of OA therapy are preventing cartilage damage and increasing subchondral bone density [23]. Recent studies have found that mechanical stimulation can promote bone formation, increase bone density, and remodel bone structure [24–26].

The MIA model has a good performance in pain related behavioral responses in the injured limb as well as significant differences between both hind limbs in the dynamic gait parameter [27]. Previous research tells that MIA at the dose of 1 mg/rat or more caused gait imbalances [28]. Although the imbalance caused by MIA at 1 mg/knee was sensitive to morphine (3 mg/kg s.c.), that caused by MIA at 1.5 mg or more was less sensitive to medication [28]. So we select the dose of 1 mg/rat as our initial choice.

The findings described here confirm the therapeutic effect of ESW in OA rats. Micro-CT findings included increased bone density, bone volume, trabecular number, and trabecular thickness (Figure 2), suggesting that ESWT promotes subchondral bone reconstruction. The gait analysis results also demonstrated that ESWT improves symptoms in OA rats (Figure 1, Figure S3). ESWT may affect the expression of molecules [29, 30] or directly the energy-related nerve fibers and then show the improvement of OA rats, and further experiments may certify this problem. Histopathologic analysis revealed that collagen in the superficial zone of subchondral bone may be repaired by ESWT, thus reducing the Mankin score of pathological changes in tissue structure. It may be better to assess chondrogenic differentiation, but we will research cartilage in the succedent experimental research. High Wnt5a protein expression in treated OA rat's subchondral bone was immunohistochemically confirmed. This suggests that ESWT exerts its beneficial effects by interfering in the transition from early- to medium-stage OA and could inhibit OA development. The goal of OA treatment is to promote the repair and reconstruction of cartilage and subchondral bone. Some groups have reported that osteogenic differentiation of BMMSCs plays pivotal roles in these processes [4, 5]. ESWT has been reported to promote cell growth and metabolism, stimulate the growth of capillaries, and enhance tissue repair [16, 31–33]. We found that Wnt5a/Ca^2+^ signaling could be activated using optimal ESW energy. Furthermore, associated gene and protein expression levels including Wnt5a, PKC, PLC, and CaMKII were influenced.

Our experiments revealed another interesting result: Low ESW energy levels may have little effect in inducing Wnt5a expression, while excessive ESWT decreased BMMSC vitality (Figure S1). Previous studies have not identified the exact optimal energy level for ESWT. We found that Wnt5a expression in treated cells peaked after a single 30 min ESWT with 0.6 bar, 6 Hz, and 1000 s. In the condition, abundant fluorescent labels for Wnt5a were observed in the perinuclear area of BMMSCs (Figure 4(d)). ESWT induced varied protein expression levels of PKC, PLC, and CaMKII, which are downstream molecules in Wnt5a/Ca^2+^ signaling pathway.

In summary, the mechanism of ESWT in OA was verified by* in vivo* and* in vitro* experiments; mechanical stimulation activated Wnt5a/Ca^2+^ signaling in BMMSCs.

## 5. Conclusion

ESWT improved symptoms in OA rats, and we confirmed that it inhibited cartilage degeneration and promoted rebuild of subchondral bone. The mechanism may be that ESWT activates Wnt5a/Ca^2+^ signaling in BMMSCs.

## Supplementary Material

The animal model and rat care were supported by Beijing Ke Yu Animal Breeding Center.

## Abbreviations

OA: Osteoarthritis
BMMSCs: Bone marrow mesenchymal stem cells
ESW: Extracorporeal shock wave
ESWT: ESW treatment
*µ*CT: Microcomputed tomography
Q-RT-PCR: Semiquantitative real-time polymerase chain reaction
SD rats: Sprague Dawley rats
BMD: Bone mineral density
BSA/BV: Bone surface area/bone volume
BV/TV: Bone volume/total volume
Tr.s: Trabecular spacing
Tr.N: Trabecular number
Tr.Th: Trabecular thickness
SEM: Standard error of mean.
