# Supplementary material for: Extracorporeal Shock Wave Rebuilt Subchondral Bone In Vivo and Activated Wnt5a/Ca2+ Signaling In Vitro
Source: Biomed Res Int. 2017 Oct 15;2017:1404650. doi: 10.1155/2017/1404650 (PMC5661093; doi:10.1155/2017/1404650)
Supplement: Supplementary file 1 — The animal model and rat care were supported by Beijing Ke Yu Animal Breeding Center. [file 1404650.f1.pdf]

Extracorporeal shock wave regenerates subchondral bone via activation of Wnt5a/Ca^{2+} signaling in an osteoarthritis rat model

Supplemental information

Method

Cell isolation and cultures

After anaesthesia, 4-week-old Sprague-Dawley rats were sacrificed and immersed in 75% alcohol for 5 minutes. BMMSCs were isolated from the bilateral femurs and tibias, and then was inoculated in the culture flask (Corning incorporated， USA) with the density of $1 \times 10^9 L^{-1}$ cells/cm^2 at the condition of 37°C, 5% CO_2. Half of the medium was changed at the first 24 hours, and then changed every three days. The BMMSCs were digested by 0.25% trypsin (Beyotime Biotechnology, China) and passaged after the area of culture bottle was covered approximately 80% to 90%. The second generation of BMMSCs was used in the experiments.

Cell counting

After the BMMSCs confluency reached to 90%, the experiment group was treated with different ESW energy levels (0.2bar, 0.4bar and 0.6bar, 1000s), and the control group were treated the same with the experiment group except ESW intervention. After treatment, BMMSCs were washed with phosphate-buffered saline（PBS）for three times, then received 500μL cell counting kit 8 each well, and were incubated for 30 minutes. 30μL supernatant was sucked out and transferred from 6-well-plate to 96-well-plate. The absorbance of 490nm was detected in the microplate reader. The number of cells was calculated according to the standard curve.

Result

ESW with 0.8 bar and 1.0 bar for 1000 shots was abandoned for treating BMMSCs

Low energy ESW had little effect on the survival of BMMSCs, whilst high energy ESW led to increased apoptosis. The DolorClast Swiss was used to generate the pneumatic ballistic shock wave, producing a 60 degree sector effective area with a radius of 4 cm (Fig.S1.B). To study the energy level of ESW *in vitro*, we first studied the effect of ESW on rat BMMSCs. ESW energy of

0.6 bar at a frequency of 1000 shots (s), was found to be safe. BMMSCs were collected and assessed for morphological changes, with no significant differences identified (3–5%, $p < 0.05$). When ESW energy was increased to 0.8 and 1.0 bar, a large number of cells detached from the culture plate and appeared unviable (Fig. S1.A). To further confirm the effects of the ESW energy range (0–1.0 bar) on cell survival, 30 min after ESW intervention, cells were stained with calcein, propidium iodide (PI) and the Cell Counting Kit-8 (CCK-8) for relative quantification (Fig.S1.A and Fig.S1.E). The results showed that at 0.8 and 1.0 bar ESW led to cell death, but at 0.2, 0.4 and 0.6 bar ESW had no effect on cell proliferation and viability.

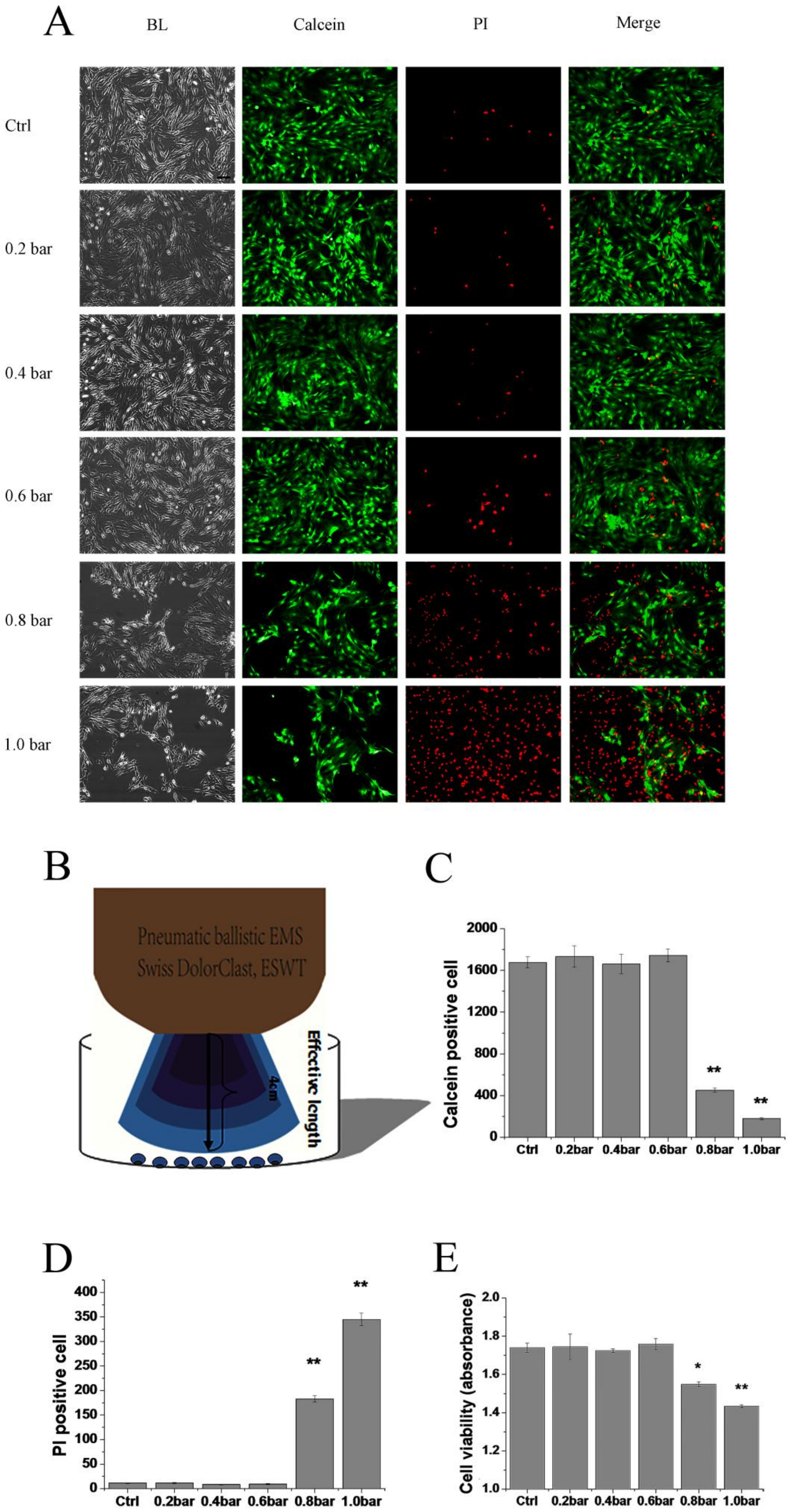

A
BL
Calcein
PI
Merge
Ctrl
0.2 bar
0.4 bar
0.6 bar
0.8 bar
1.0 bar
B
Pneumatic ballistic EMS
Swiss DolorClast, ESWT
Effective length
4cm
C
Calcein positive cell
Ctrl 0.2bar 0.4bar 0.6bar 0.8bar 1.0bar
D
PI positive cell
Ctrl 0.2bar 0.4bar 0.6bar 0.8bar 1.0bar
E
Cell viability (absorbance)
Ctrl 0.2bar 0.4bar 0.6bar 0.8bar 1.0bar

Fig.S1. Quantitative cell viability assay in the absence and presence of ESW stimulation.. (A) Effect of ESW energy level on BMMSC survival. Images were taken under different conditions (gray images: bright light; green: calcein staining, and red: PI staining). Scale bar = 100 µm. (B) Schematic description of the ESW stimulation assay. Cell culture medium (10% FBS) height close to the upper edge of the plate. The shock wave probe just touched the liquid of the plate and vertical interference was performed on the cells. The effective radius of the shock wave in the liquid was 4 cm. (C) Quantitative calcein staining for cell viability. BMMSCs were seeded into plates. Thirty minutes after ESW intervention, calcein or PI stain was added, and cells were counted under a fluorescence microscope using a ×10 objective. Histogram shows the calcein-positive cell number. (D) Histogram shows the PI-positive cell number. (E) Cell relative activity assay. CCK-8 was used to detect the relative activity of the cells. *p < 0.05 and **p < 0.01 compared with control (error bar is SEM).

Table S1. Mankin score of OA pathological grading

**Score**	**Tissue structure**	**Cells**	**Matrix staining**	**Structural integrity of tide line**
0	normal	normal	normal	integral
1	irregular surface	cell increased diffusely	mild reduction	broken
2	pannus and irregular surface	local cells increased	moderate reduction	
3	cracks in the transition zone	cell apoptosis	severe reduction	
4	cracks in the radiation belt		not colored	
5	cracks in the calcified zone			

6	structure completely destroyed

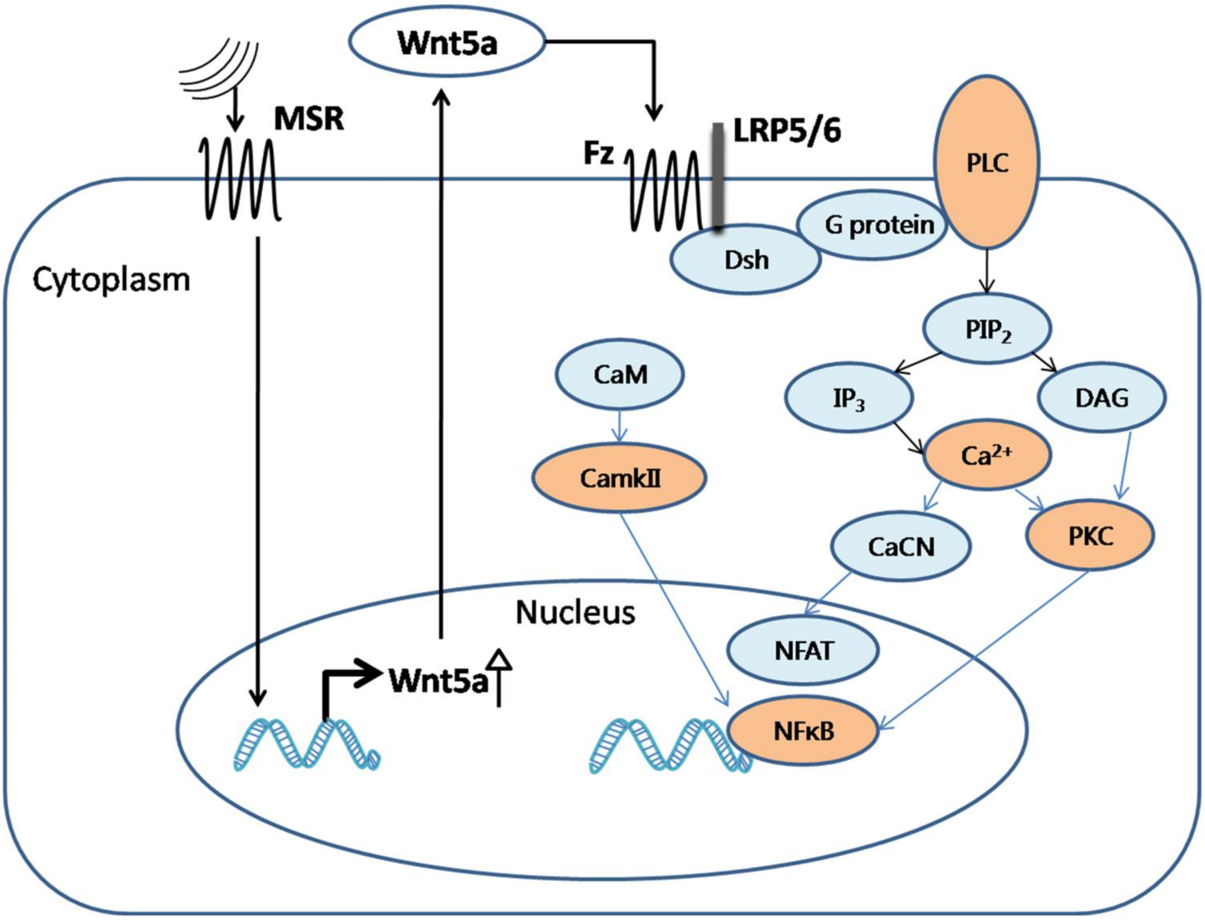

Fig.S2. Proposed model for the Wnt5a/Ca2+ signaling pathway in moderating expression of related proteins. Expression of Wnt5a is up-regulated following the activation of mechanically sensitive receptors on the cell surface. Wnt5a protein then combines with disheveled (Dsh), a member of the frizzled (Fz) family, by an autocrine or paracrine mechanism. This in turn causes the intracellular Ca2+ concentration to increase which activates PKC, PLC and CamkII. This induces activation of NFAT and NF-κB through a series of processes, which initiates gene transcription of corresponding sequences.

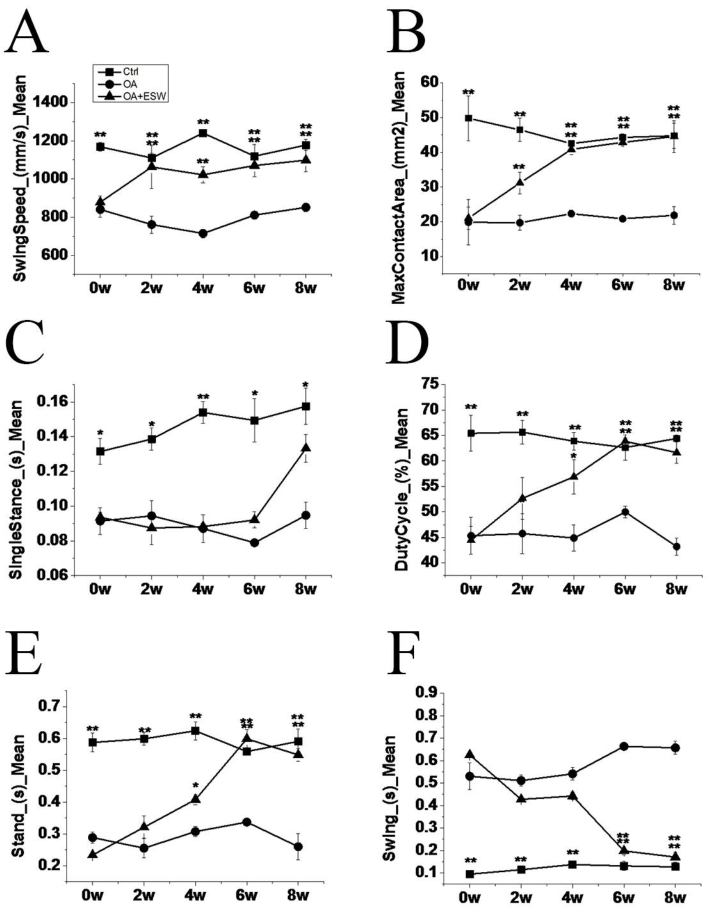

Fig.S3. Gait parameters obtained from the CatWalk analysis for each group. Some results were show at Fig.1. And the data above was additional. Values presented are mean ± 95% confidence interval of the corresponding numerical. There is no difference between OA and OA+ESW in the gait parameters of Single Stance(n=12, * P<0.05). With the OA group significant differences are present for all the other five gait parameters (n=12, * P<0.05) except for Duty Cycle and Stand on 0w and 2w, Max Contact Area on 0w and Swing on 0w, 2w and 4w. Error bar is standard error of mean [SEM].
